# Supplementary material for: Dirichlet-Swing: understanding spatio-temporal aspects of political elections in heterogeneous societies through agent-based simulation
Source: PLoS One. 2026 Mar 17;21(3):e0344018. doi: 10.1371/journal.pone.0344018 (PMC12994849; doi:10.1371/journal.pone.0344018)
Supplement: S1 File — (PDF) [file pone.0344018.s001.pdf]

Supporting Information for 'Dirichlet-Swing: understanding spatio-temporal aspects of political elections in heterogeneous societies through agent-based simulation'

A Additional Analyses and Results

A.1 Comparison of Swing Models

We have proposed the Dirichlet Swing Model (DSM) and the Dirichlet Swing Matrix Model (DSMM) to simulate the process of vote swings between successive elections. For each of the elections simulated by DPM, PCM, SIM in the previous analysis (referred to as DPM-1, PCM-2 etc in Tables 1 and 2 of the main paper), we simulated the *next election* using both the DSM and DSMM. We consider different values of the swing $\Delta\theta(t)$. The base distribution parameters ν is chosen as $B\Delta\hat{\theta}(t)$, where hyperparameter B controls the variance across the atoms, i.e. district-wise swings. In each case, we estimate the new vote share and the corresponding seat share. The results are shown in Table 9. We also count the *seat flips* - the number of seats that change hands across the successive elections. We find that the number of seat flips is sometimes greater than the absolute seat differences of the different parties - suggesting that even a party that loses votes may gain new seats under our model, which is impossible under the Uniform Swing and Proportional Swing models.
The tabulated results in Table 1, however, indicate that the two models of swing provide quite contradictory results. For example, the first and fifth rows indicate the largest party (P1) losing some votes, while the second and third party gain almost equally. However, while DSM model suggests a big loss of P1 and a significant gain for the smallest party P3 in terms of seats, DSMM suggests a smaller loss for P1 and very little gains for P3. However, both models agree that P2 gains significantly. The difference is best understood by considering the number of flips, which is generally higher in DSM. However, the results of DSMM are more aligned with DPM model.

A.2 Spatial variance of vote shares

The key reason why the most popular party (highest vote share) does not win all the seats is the variance of vote shares across districts. Higher this variance, more diverse the results in terms of seat distribution. In Table 2, we report the standard deviation of the votes secured by each party across the districts, in the actual as well as simulated versions of the elections. We see that in most cases, the elections simulated by DPM under optimal parameter settings are quite similar to the actual elections in terms of standard deviation, though PCM tends to exaggerate the standard deviation in some cases. Understandably, the standard deviation is lower under the Default Parameter Setting 1, compared to Default Parameter setting 2, as the latter promotes spatial concentration of voters of any party, resulting in higher variance across districts.

Table 1. Changes in Vote Shares and Seat Shares between Successive Elections using District Swing Model (DSM) and District Swing Matrix Model (DSMM)

$X^1(t)$	$X^2(t)$	Swing(B)	$X^1(t+1)$	$X^2(t+1)$	Flips
[0.45,0.35,0.2]	[0.74,0.23,0.03]	DSM(10)	[0.4,0.38,0.22]	[0.43,0.4,0.17]	47
[0.45,0.35,0.2]	[0.74,0.23,0.03]	DSM(100)	[0.4,0.38,0.22]	[0.5,0.44,0.06]	29
[0.45,0.35,0.2]	[0.74,0.23,0.03]	DSM(10)	[0.48,0.27,0.25]	[0.62,0.18,0.20]	39
[0.45,0.35,0.2]	[0.74,0.23,0.03]	DSM(100)	[0.48,0.27,0.25]	[0.83,0.1,0.07]	20
[0.45,0.35,0.2]	[0.74,0.23,0.03]	DSMM(10)	[0.41,0.37,0.22]	[0.55,0.41,0.04]	22
[0.45,0.35,0.2]	[0.74,0.23,0.03]	DSMM(100)	[0.41,0.37,0.22]	[0.56,0.41,0.03]	20
[0.45,0.35,0.2]	[0.74,0.23,0.03]	DSMM(10)	[0.47,0.28,0.25]	[0.87,0.08,0.05]	22
[0.45,0.35,0.2]	[0.74,0.23,0.03]	DSMM(100)	[0.47,0.28,0.25]	[0.92,0.05,0.03]	18

Table 2. Standard Deviation of vote shares of the parties across districts in actual elections, and the simulations by DPM and PCM under default and optimal parameter settings (values of standard deviation expressed as multiples of 10^4)

Election	Model	Opt. Param.			Def. Param. 1			Def. Param. 2		
		σ_1	σ_2	σ_3	σ_1	σ_2	σ_3	σ_1	σ_2	σ_3
GJ17	Actual	2.26	1.88	1.38	x	x	x	x	x	x
GJ17	DPM	2.82	2.56	2.35	1.26	1.11	1.36	3.53	3.33	2.73
GJ17	PCM	5.5	5.1	1.8	4.0	3.77	1.28	5.5	5.1	1.8
GJ22	Actual	2.78	2.34	2.01	x	x	x	x	x	x
GJ22	DPM	3.02	2.11	2.16	x	x	x	x	x	x
GJ22	PCM	3.3	2.3	2.1	0.46	0.29	0.18	5.46	4.1	2.06
WB19	Actual	2.1	1.9	2.5	x	x	x	x	x	x
WB19	DPM	1.88	2.06	1.77	1.31	1.51	1.23	3.6	3.4	3.6
WB19	PCM	3.53	2.29	3.31	0.42	0.19	0.41	5.9	2.3	5.7
WB21	Actual	1.85	1.4	2.4	x	x	x	x	x	x
WB21	DPM	2.2	2.03	1.89	1.59	1.58	1.24	4.44	3.14	4.08
WB21	PCM	3.23	0.47	4.29	0.5	0.14	0.41	6.38	1.38	5.84

A.3 Role of sampling parameters in Survey Projections

Next, we consider the importance of spatial coverage f_s and person coverage f_n. In general, we can expect that if there is significant diversity in terms of vote share across the districts, performance should improve if we consider more districts in our survey. But if such diversity does not exist, then surveying more districts (high f_s) has no advantage. Similarly, if the vote shares of different parties are close to each other then sampling more voters (high f_n) can improve the estimates, but this does may not be true if the vote shares are well-separated.

We consider i) four elections simulated by the GDPM model with different levels of popular support and the concentration parameter α, ii) four elections simulated by G-PCM with different levels of popular support and the concentration parameter η, iii) 2 elections simulated by SIM (one where different communities have comparable preferences, and one where different communities have markedly different preferences). The full results in tabular form are provided Tables 11,12,13. We find that there is no straightforward relation between projection performance and f_n or f_s. Performance tends to improve with f_s in case of the elections simulated by DPM and PCM, but less so in case of SIM. The reverse is true in case of f_n. The projection performance generally tends to be significantly worse in case of the elections simulated by SIM, as it is a more sophisticated model capable of adding more layers of uncertainty through community-based preferences of voters.

Table 3. Change in seat projections due to variation of f_s, f_n on elections simulated by G-DPM (upper part: DPM-6, lower part: DPM-7). We report the mean Manhattan Distance in each case, while the probability of accurate projection is shown in brackets.

f_n/f_s	0.1	0.25	0.5	1.0
0.001	0.30 (0.11)	0.18 (0.09)	0.12 (0.12)	0.08 (0.21)
0.005	0.32 (0.13)	0.18 (0.07)	0.12 (0.16)	0.05 (0.55)
0.01	0.30 (0.12)	0.18 (0.05)	0.11 (0.16)	0.05 (0.56)
0.05	0.28 (0.12)	0.18 (0.05)	0.11 (0.06)	0.04 (0.74)
0.1	0.27 (0.15)	0.18 (0.07)	0.10 (0.18)	0.03 (0.86)
0.001	0.29 (0)	0.20 (0.02)	0.15 (0.05)	0.15 (0.05)
0.005	0.29 (0)	0.19 (0.03)	0.12 (0.09)	0.07 (0.29)
0.01	0.31 (0)	0.17 (0.06)	0.10 (0.16)	0.06 (0.43)
0.05	0.29 (0)	0.18 (0.03)	0.10 (0.14)	0.03 (0.85)
0.1	0.31 (0)	0.15 (0.04)	0.1 (0.12)	0.02 (0.93)

Table 4. Change in seat projections due to variation of f_s, f_n on elections simulated by G-PCM (upper part: PCM-6, lower part: PCM-9). We report the mean Manhattan Distance in each case, while the probability of accurate projection is shown in brackets.

f_n/f_s	0.1	0.25	0.5	1.0
0.001	0.31 (0)	0.20 (0.03)	0.12 (0.13)	0.04 (0.61)
0.005	0.33 (0)	0.19 (0.01)	0.11 (0.14)	0.03 (0.86)
0.01	0.36 (0)	0.17 (0.08)	0.10 (0.18)	0.02 (0.92)
0.05	0.33 (0)	0.19 (0.07)	0.10 (0.16)	0.02 (0.98)
0.1	0.33 (0)	0.19 (0.07)	0.10 (0.12)	0.01 (1.00)
0.001	0.35 (0)	0.17 (0.04)	0.12 (0.15)	0.07 (0.34)
0.005	0.32 (0)	0.20 (0.04)	0.11 (0.11)	0.04 (0.65)
0.01	0.32 (0)	0.21 (0.01)	0.11 (0.13)	0.03 (0.86)
0.05	0.31 (0)	0.20 (0.03)	0.11 (0.12)	0.02 (0.99)
0.1	0.34 (0)	0.19 (0.03)	0.12 (0.08)	0.01 (1.00)

Table 5. Change in seat projections due to variation of f_s, f_n on elections simulated by SIM (upper part SIM-1, lower part SIM-2). We report the mean Manhattan Distance in each case, while the probability of accurate projection is shown in brackets.

f_n/f_s	0.1	0.25	0.5	1.0
0.001	0.42 (0)	0.43 (0)	0.49 (0)	0.56 (0)
0.005	0.32 (0)	0.23 (0.02)	0.27 (0)	0.36 (0)
0.01	0.30 (0)	0.21 (0.04)	0.19 (0.02)	0.25 (0)
0.05	0.24 (0)	0.16 (0.13)	0.12 (0.07)	0.1 (0.1)
0.1	0.24 (0)	0.15 (0.1)	0.10 (0.18)	0.07 (0.31)
0.001	0.32 (0)	0.27 (0.02)	0.26 (0.01)	0.26 (0.04)
0.005	0.32 (0)	0.20 (0.05)	0.16 (0.04)	0.16 (0.04)
0.01	0.34 (0)	0.20 (0.08)	0.15 (0.12)	0.12 (0.15)
0.05	0.33 (0)	0.20 (0.03)	0.13 (0.15)	0.07 (0.32)
0.1	0.34 (0)	0.20 (0.03)	0.13 (0.07)	0.07 (0.33)
