# Supplementary material for: Dirichlet-Swing: understanding spatio-temporal aspects of political elections in heterogeneous societies through agent-based simulation
Source: PLoS One. 2026 Mar 17;21(3):e0344018. doi: 10.1371/journal.pone.0344018 (PMC12994849; doi:10.1371/journal.pone.0344018)
Supplement: Attachment — Submitted filename: PLoS_rebuttal2.pdf [file pone.0344018.s004.pdf]

Rebuttal Letter for "Dirichlet-Swing: understanding spatio-temporal aspects of political elections in heterogeneous societies through agent-based simulation"

I thank the Reviewers and the Academic Editor for their time and effort in reviewing my paper. I have highlighted the newly added text in green colour, and indicated removed text by striking through.

Comments by Reviewer 1: I thank Reviewer 1 for their favorable assessments.

Comments by Reviewer 2: The authors created graphs comparing the described and simulated models, but the graphs are of very poor quality. They are very difficult to read; they seem blurry. In order to make things easier for the reader, in the previous comments I suggested a flowchart for the models. It may not have been described in great detail, but in my opinion, a block diagram was best, showing which model contains which assumptions, so that it would be easy to understand the differences between the models.

Response: I have replaced the previous images to improve their sharpness and quality using the PACE tool of PLOS One. I have also added two new figures – Fig 1: a block diagram showing the workflow of the paper, indicating the roles of the different models including their inputs and outputs, and Fig 2: a table listing the different models along with their inputs, outputs, parameters and assumptions.
