# Supplementary material for: Dirichlet-Swing: understanding spatio-temporal aspects of political elections in heterogeneous societies through agent-based simulation
Source: PLoS One. 2026 Mar 17;21(3):e0344018. doi: 10.1371/journal.pone.0344018 (PMC12994849; doi:10.1371/journal.pone.0344018)
Supplement: Attachment — Submitted filename: PLoS_rebuttal.docx [file pone.0344018.s003.docx]

**Rebuttal Letter for “Dirichlet-Swing: understanding spatio-temporal aspects of political elections in heterogeneous societies through agent-based simulation”**

**Comments by Academic Editor:**

**Comment**: I recommend that the authors take into account the remarks of reviewer 2, especially remarks related to introducing graphics that may simplify cross-comparisons between models.

**Response**: I already had 3 figures that demonstrate the spatial maps of district-based elections simulated by the models discussed. In addition, I have added plots to compare vote share vs seat share (Figures 4 and 5) in the proposed swing models. I have also replaced the tabular presentation of the results related to the impact of ‘district coverage’ and ‘person coverage’ parameters on the accuracy of election surveys, using plots for the same (Figure 6-8). I hope these graphics will simplify the visualization of the results.

**Comment:** Regarding the issue of social identity and voting behaviour, there are few studies that the authors may find useful - I send them few suggestions, in case they think they have relevance in this context of the literature review of this study ([doi.org/10.1371/journal.pone.0331959](http://doi.org/10.1371/journal.pone.0331959) & [doi.org/10.1016/j.physa.2023.128675](http://doi.org/10.1016/j.physa.2023.128675)).

**Response**: I thank the editor for these suggestions; they are indeed relevant to this study. I have cited both of these papers, and also discussed the position of this paper with respect to them lines (215-218) and (316-319).

**Comment**: Another issue raised by reviewers is the length of the manuscript. I suggest a concise writing and that redundancies be summarised to make the paper more readable and accessible for a broad audience.

**Response:** I have removed some experimental results which I feel are less important for the main premise of the paper to an “Appendix” section. These include the complete tabular data related to simulation of swings by the proposed DSM and DSMM models (graphical description of the results has already been provided in Figures 4 and 5 in the main text). The results about spatial variation of the vote shares, and the full tabular data related to the variation of survey results based on survey parameters have also been moved to the “Appendix”. I am not sure if PLoS One allows “Appendix” sections – if not, this section can be dropped altogether. Additionally, I have modified the text to make it more concise wherever possible.

**Comments by Reviewer 1:** This manuscript presents a technically ambitious and original contribution to the study of district-based elections. It integrates agent-based modeling, Monte Carlo simulation, and Dirichlet Process–based statistical inference to examine how spatial heterogeneity, social identity, and temporal vote swings shape electoral outcomes. The study’s main contribution lies in introducing Dirichlet-based swing models for electoral dynamics, which extend existing statistical approaches to vote transitions by offering a more nuanced treatment of spatial and temporal heterogeneity. This interdisciplinary framework bridges political science, statistics, and computational modeling, providing a scalable method capable of simulating real-world multiparty elections with greater realism than conventional swing models. Moreover, it opens promising avenues for integrating survey simulations and predictive analytics in heterogeneous societies, marking an innovative direction with strong cross-disciplinary potential.

**Response**: I thank Reviewer 1 for their positive assessment of the work.

**Comments by Reviewer 2**: In this work, the authors compare multiple election models. Finally, they use the models to reconstruct the election results in India. The authors propose two new models. The work is very extensive and references numerous works of literature. It would greatly benefit from the addition of graphics comparing models, model features, etc., as well as from greater emphasis on where the author's model is discussed. Other methods that would improve the readability of the work were also highly recommended. Works that compare methods/models are very necessary, but in such works, their usefulness to the reader is even more important. The authors should overcomplicate the work, perhaps by shortening it and making it more attractive with graphics, but also by making it more user-friendly.

**Response**: I thank Reviewer 2 for their assessment of the work and the suggestions. I have addressed the suggestions in my response to the Academic Editor.

**Other issues**: I have made sure that all tables and figures provided have been referenced in the text. I have used the PLoS One Latex Template, and followed the formatting instructions as far as possible.
